# Supplementary material for: Proteomics Reveals mRNA Regulation and the Action of Annexins in Thyroid Cancer
Source: Int J Mol Sci. 2023 Sep 26;24(19):14542. doi: 10.3390/ijms241914542 (PMC10572572; doi:10.3390/ijms241914542)
Supplement: Supplementary file 1 [file ijms-24-14542-s001.zip › ijms-2624618-supplementary.pdf]

Proteomics reveals mRNA regulation and the action of annexins in thyroid cancer

Supplementary material

Tables

Table S1. Discrimination of the thyroid nodules of the entire studied population. Diagnosis by fine-needle aspiration biopsy (FNAB) cytology [1] and histology diagnosis [2] of all samples, including subtypes in parenthesis. Plus, discrimination of the thyroid nodules used for HR-MAS ^{1}H NMR metabolomics. Abbreviations: AUS - atypia of undetermined significance; BT – benign tumours; FTC – follicular thyroid carcinoma; PTC – papillary thyroid carcinoma.

Individual	Sample	Cytology diagnosis	Histology diagnosis	HR-MAS ^{1}H NMR
TR008	A	AUS	BT (Folicular adenoma)	
TR012	A	Benign	BT (Follicular nodular disease)	✓
	B	Benign	BT (Follicular nodular disease)	
TR013	A	Follicular tumour	PTC (Follicular)	✓
TR014	A	Benign	BT (Follicular nodular disease)	
	B		BT (Follicular nodular disease)	
	C		BT (Follicular nodular disease)	
TR015	A		BT (Follicular nodular disease)	
	B		BT (Follicular nodular disease)	
TR018	A	Benign	BT (Follicular nodular disease)	✓
TR019	A	Benign	BT (Follicular nodular disease)	
	B		BT (Follicular nodular disease)	
TR020	A	Follicular tumour (oncocytic)	FTC (Oncocytic)	✓
TR021	A	Benign	BT (Follicular adenoma)	✓
	B	AUS	BT (Follicular nodular disease)	
TR023	A	Benign	BT (Follicular nodular disease)	
TR025	A		BT (Follicular nodular disease)	
	B		BT (Follicular nodular disease)	
	C	Benign	BT (Follicular nodular disease)	
TR026	A	Follicular tumour	BT (Follicular nodular disease)	✓
	B	Benign	BT (Follicular nodular disease)	
TR028	A	Follicular tumour	PTC (Follicular and solid)	✓
TR029	A	Benign	BT (Follicular nodular disease)	
TR030	A		BT (Follicular nodular disease)	
TR031	A	Benign	BT (Follicular nodular disease)	✓
	B	Follicular tumour	BT (Follicular nodular disease)	
TR032	A	Follicular tumour	PTC (Follicular)	✓
TR034	A	Papillary carcinoma	PTC (Classical and follicular)	✓
	B		PTC (Classical and follicular)	
TR035	A	Benign	BT (Follicular nodular disease)	
TR037	A		BT (Follicular nodular disease)	✓
	B		BT (Follicular nodular disease)	
	C		BT (Follicular nodular disease)	
	D		BT (Follicular nodular disease)	
TR038	A	Non-diagnostic	BT (Follicular adenoma)	✓
	B		BT (Follicular nodular disease)	
TR039	A	Benign	BT (Follicular nodular disease)	✓
TR040	A	Benign	BT (Follicular nodular disease)	✓
	B		BT (Follicular nodular disease)	
TR041	A	Benign	BT (Follicular nodular disease)	
TR043	A	Benign	BT (Follicular nodular disease)	
	B		BT (Follicular nodular disease)	
	C	Benign	BT (Follicular nodular disease)	
	D		BT (Follicular nodular disease)	
TR045	A	Non-diagnostic	BT (Follicular nodular disease)	

	B		BT (Follicular nodular disease)	
TR046	A	Benign	BT (Follicular nodular disease)	
	B	Benign	BT (Follicular nodular disease)	
TR047	A		BT (Follicular nodular disease)	✓
	B	Benign	BT (Follicular nodular disease)	
TR048	A	Benign	BT (Follicular nodular disease)	✓
TR049	A	Benign	BT (Follicular nodular disease)	
TR050	A	Papillary carcinoma	PTC (Classical and follicular)	✓
TR051	A	Papillary carcinoma	PTC (Classical)	✓
TR052	A	Benign	BT (Follicular nodular disease)	
	B	Benign	BT (Follicular nodular disease)	
TR053	A	Papillary carcinoma	PTC (Classical)	✓
TR054	A	Papillary carcinoma	PTC (Diffuse sclerosing)	✓
TR055	A	Papillary carcinoma	PTC (Classical)	✓
TR056	A	Benign	BT (Follicular nodular disease)	✓
	B	Benign	BT (Follicular nodular disease)	
TR057	A	Non-diagnostic	BT (Follicular nodular disease)	✓
TR058	A	Non-diagnostic	BT (Follicular nodular disease)	
	B		BT (Follicular nodular disease)	
TR059	A	Benign	BT (Follicular nodular disease)	
TR060	A	Benign	BT (Follicular nodular disease)	
	B	Benign	BT (Follicular nodular disease)	
	C	Benign	BT (Follicular nodular disease)	
TR061	A	Benign	BT (Follicular nodular disease)	
	B	Benign	BT (Follicular nodular disease)	
TR063	A	Oncocytic adenoma	BT (Oncocytic adenoma)	
Individuals	43	Non-diagnostic 4<br>AUS 2	Inadequate or different from cytology 8 (15.7%)	

Table S2. Enriched Reactome pathways by thyroid tissue malignancy. List of top Reactome pathways ranked by p-value, with at least 20 entities total and 25% entities found. "Entities found/total" correspond to the number of proteins in the submitted list that belong to each pathway, followed by the total number of proteins for that pathway; "% entities/pathway" is the percentage of entities of this dataset found in each pathway; "p-value" is the results of PADOG method to determine gene enrichment; "FDR" is the false discovery rate of these attributions and "Regulation" informs whether this pathway is up- or down-regulated.

Reactome pathway	Entities found/ total	% entities/ pathway	p-value	FDR	Regulation
Neutrophil degranulation	234/480	48.8	0.001	0.148	Up
Glutathione conjugation	18/37	48.6	0.006	0.372	Down
Innate Immune System	376/1201	31.3	0.009	0.505	Up
RHOV GTPase cycle	11/37	29.7	0.009	0.505	Up
Post-translational protein phosphorylation	46/107	43.0	0.01	0.505	Down
Metabolism of carbohydrates	101/300	33.7	0.011	0.528	Down
Interleukin-12 signaling	30/46	65.2	0.012	0.549	Down
RHO GTPases activate IQGAPs	13/32	40.6	0.014	0.56	Up
Gene and protein expression by JAK-STAT signaling after Interleukin-12 stimulation	29/37	78.4	0.016	0.591	Down
Interleukin-12 family signaling	33/56	58.9	0.016	0.591	Down
Biological oxidations	56/223	25.1	0.016	0.591	Down
Phase II - Conjugation of compounds	32/111	28.8	0.017	0.616	Down
Regulation of Insulin-like Growth Factor (IGF) transport and uptake by Insulin-like Growth Factor Binding Proteins (IGFBPs)	50/124	40.3	0.018	0.64	Down
Neurodegenerative Diseases	11/22	50.0	0.019	0.64	Up
Deregulated CDK5 triggers multiple neurodegenerative pathways in Alzheimer's disease models	11/22	50.0	0.019	0.64	Up
Defective Intrinsic Pathway for Apoptosis	12/25	48.0	0.021	0.66	Up
Gluconeogenesis	19/34	55.9	0.024	0.688	Down
RA biosynthesis pathway	6/22	27.3	0.024	0.688	Down
Intrinsic Pathway of Fibrin Clot Formation	14/23	60.9	0.029	0.752	Down
Signaling by Interleukins	130/470	27.7	0.032	0.798	Up
Platelet degranulation	70/128	54.7	0.033	0.802	Down
Detoxification of Reactive Oxygen Species	23/39	59.0	0.035	0.817	Down
Response to elevated platelet cytosolic Ca^{2+}	71/133	53.4	0.036	0.817	Down
Platelet activation, signaling and aggregation	99/265	37.4	0.036	0.817	Down
Nuclear Envelope (NE) Reassembly	34/78	43.6	0.043	0.907	Up
Nicotinate metabolism	10/31	32.3	0.044	0.918	Up

Adherens junctions interactions	9/33	27.3	0.049	0.952	Down
IRE1alpha activates chaperones	19/50	38.0	0.049	0.952	Down

Table S3. Biological processes altered by malignancy according to the Gorilla web application. The *P*-value corresponds to the enrichment *p*-value computed according to the minimum hypergeometric (mHG) or HG model; the FDR q value is the correction of the above *p*-value for multiple testing using the Benjamini and Hochberg method.

**Biological process**	***P*-value**	**FDR q value**
nucleic acid metabolic process	7.27E-14	6.68E-10
viral process	3.58E-13	1.64E-09
symbiont process	3.58E-13	1.10E-09
interspecies interaction between organisms	8.47E-13	1.95E-09
RNA metabolic process	9.04E-12	1.66E-08
macromolecule localization	1.73E-10	2.65E-07
multi-organism process	1.88E-10	2.46E-07
establishment of protein localization	3.73E-10	4.29E-07
nitrogen compound transport	5.03E-10	5.14E-07
ncRNA metabolic process	5.64E-10	5.18E-07
mRNA metabolic process	5.72E-10	4.78E-07

Table S4. Enriched KEGG pathways by thyroid tissue malignancy. Ranked list of KEGG pathways according to DAVID. Count corresponds to the number of proteins in the submitted list that belong to each pathway; "%" is the percentage of proteins in each pathway relative to the total of imported proteins; p-value is the result of Fisher's Exact test to determine gene enrichment; and FDR is the false discovery rate of these attributions.

KEGG pathway	Count	% entities/ pathway	*p*-value	FDR
Ribosome	71	4.3	<0.001	<0.001
Spliceosome	63	3.8	<0.001	<0.001
Proteasome	29	1.8	<0.001	<0.001
Coronavirus disease - COVID-19	73	4.4	<0.001	<0.001
Prion disease	80	4.9	<0.001	<0.001
Carbon metabolism	46	2.8	<0.001	<0.001
Amyotrophic lateral sclerosis	96	5.8	<0.001	<0.001
Huntington disease	84	5.1	<0.001	<0.001
Nucleocytoplasmic transport	41	2.5	<0.001	<0.001
Citrate cycle (TCA cycle)	20	1.2	<0.001	<0.001
Parkinson disease	73	4.4	<0.001	<0.001
Salmonella infection	65	4	<0.001	<0.001
Protein processing in endoplasmic reticulum	49	3	<0.001	<0.001
Valine, leucine and isoleucine degradation	21	1.3	<0.001	<0.001
Diabetic cardiomyopathy	52	3.2	<0.001	<0.001
Pathways of neurodegeneration - multiple diseases	97	5.9	<0.001	<0.001
Phagosome	42	2.6	<0.001	<0.001
Alzheimer disease	82	5	<0.001	<0.001
Endocytosis	58	3.5	<0.001	<0.001
Systemic lupus erythematosus	37	2.3	<0.001	<0.001
Pathogenic Escherichia coli infection	48	2.9	<0.001	<0.001
Aminoacyl-tRNA biosynthesis	23	1.4	<0.001	<0.001
Regulation of actin cytoskeleton	53	3.2	<0.001	<0.001
Fatty acid metabolism	20	1.2	<0.001	<0.001
Oxidative phosphorylation	35	2.1	<0.001	<0.001
mRNA surveillance pathway	28	1.7	<0.001	<0.001
Bacterial invasion of epithelial cells	24	1.5	<0.001	0.001
Chemical carcinogenesis - reactive oxygen species	50	3	<0.001	0.001
Focal adhesion	46	2.8	<0.001	0.001
Shigellosis	53	3.2	<0.001	0.001
Fatty acid degradation	16	1	<0.001	0.001
Spinocerebellar ataxia	35	2.1	<0.001	0.002
Lysosome	32	1.9	<0.001	0.003
Pyruvate metabolism	16	1	<0.001	0.004
RNA degradation	22	1.3	0.001	0.005
Biosynthesis of amino acids	21	1.3	0.001	0.006
Viral carcinogenesis	43	2.6	0.001	0.007
Viral myocarditis	18	1.1	0.001	0.007
Protein export	10	0.6	0.001	0.009
Propanoate metabolism	12	0.7	0.001	0.009
Metabolic pathways	232	14.1	0.002	0.011
Fc gamma R-mediated phagocytosis	24	1.5	0.002	0.013

ECM-receptor interaction	22	1.3	0.003	0.017
Tuberculosis	37	2.3	0.003	0.021
Glutathione metabolism	16	1	0.004	0.024
Viral life cycle - HIV-1	17	1	0.004	0.026
Complement and coagulation cascades	21	1.3	0.005	0.027
Sulfur metabolism	6	0.4	0.005	0.027
Adherens junction	18	1.1	0.006	0.036
2-Oxocarboxylic acid metabolism	8	0.5	0.007	0.036
Antigen processing and presentation	19	1.2	0.008	0.041
Neutrophil extracellular trap formation	37	2.3	0.008	0.043
Glycolysis / Gluconeogenesis	17	1	0.008	0.043
Glyoxylate and dicarboxylate metabolism	10	0.6	0.010	0.050
Tight junction	33	2	0.012	0.061
Pertussis	18	1.1	0.013	0.063
Non-homologous end-joining	6	0.4	0.017	0.084
Nucleotide metabolism	19	1.2	0.018	0.088
Epstein-Barr virus infection	37	2.3	0.020	0.096
Legionellosis	14	0.9	0.024	0.110
Leukocyte transendothelial migration	23	1.4	0.028	0.130
Influenza A	31	1.9	0.039	0.170
Type I diabetes mellitus	11	0.7	0.039	0.170
Drug metabolism - other enzymes	17	1	0.041	0.180
Arginine and proline metabolism	12	0.7	0.045	0.190
Fatty acid elongation	8	0.5	0.047	0.190
Fructose and mannose metabolism	9	0.5	0.050	0.200
Proteoglycans in cancer	35	2.1	0.059	0.240
Proximal tubule bicarbonate reclamation	7	0.4	0.062	0.250
Necroptosis	28	1.7	0.067	0.260
Other glycan degradation	6	0.4	0.068	0.260
Fatty acid biosynthesis	6	0.4	0.068	0.260
Tryptophan metabolism	10	0.6	0.077	0.290
Nicotinate and nicotinamide metabolism	9	0.5	0.077	0.290
Thermogenesis	38	2.3	0.081	0.300
Amino sugar and nucleotide sugar metabolism	11	0.7	0.084	0.310
Human immunodeficiency virus 1 infection	35	2.1	0.086	0.310
beta-Alanine metabolism	8	0.5	0.089	0.310
Asthma	8	0.5	0.089	0.310
Apoptosis	24	1.5	0.089	0.310
Peroxisome	16	1	0.090	0.310
HIF-1 signaling pathway	20	1.2	0.091	0.310
Vibrio cholerae infection	11	0.7	0.094	0.310
Epithelial cell signaling in Helicobacter pylori infection	14	0.9	0.099	0.320
Platelet activation	22	1.3	0.100	0.320
Allograft rejection	9	0.5	0.100	0.320

Table S5. Proteins with AUC>0.95 for univariate ROC analysis. Mean values are derived from relative abundance quantifications. SEM – Standard error of the mean, AUC – Area under the ROC curve

Protein	Benign mean ± SEM	Malignant mean ± SEM	Log2 median fold change	Mann-Whitney p-value	AUC
P04083|ANXA1	0.000917±0.000075	0.007239±0.001537	2.44	< 0.0001	0.99
P08727|K1C19	0.000213±0.000058	0.003353±0.000954	4.24	< 0.0001	0.98
P50995|ANX11	0.000255±0.000015	0.000693±0.000064	1.58	< 0.0001	0.98
Q9UKK3|PARP4	0.000006±0.000001	0.00003±0.000004	2.25	< 0.0001	0.98
Q9UPN3|MACF1	0.000045±0.000002	0.000101±0.000009	1.28	< 0.0001	0.97
Q9NZM1|MYOF	0.000123±0.000007	0.000348±0.000042	1.44	< 0.0001	0.97
Q15149|PLEC	0.000461±0.000035	0.001403±0.000149	1.70	< 0.0001	0.97
Q99536|VAT1	0.000435±0.000022	0.000894±0.000057	1.09	< 0.0001	0.97
P06753|TPM3	0.000063±0.000004	0.000248±0.000036	1.70	< 0.0001	0.97
P02763|A1AG1	0.000235±0.00003	0.000023±0.000004	-3.02	< 0.0001	0.97
Q9BUF5|TBB6	0.000079±0.000004	0.000216±0.000028	1.35	< 0.0001	0.97
Q6UX53|MET7B	0.000005±0.000001	0.000119±0.000049	4.21	< 0.0001	0.97
O60831|PRAF2	0.000021±0.000001	0.000054±0.000005	1.41	< 0.0001	0.97
Q14195|DPYL3	0.000194±0.000015	0.000864±0.00016	2.17	< 0.0001	0.97
Q9BSJ8|ESYT1	0.000169±0.000012	0.000426±0.00004	1.36	< 0.0001	0.96
Q9BTV4|TMM43	0.000069±0.000007	0.00024±0.000026	1.95	< 0.0001	0.96
P46821|MAP1B	0.000028±0.000003	0.000096±0.000012	2.09	< 0.0001	0.96
P02545|LMNA	0.002557±0.000144	0.00588±0.000561	1.28	< 0.0001	0.96
Q9Y646|CBPQ	0.007057±0.000618	0.001622±0.000386	-2.84	< 0.0001	0.96
Q9P2R3|ANFY1	0.000009±0.000001	0.000018±0.000002	0.92	< 0.0001	0.96
Q13509|TBB3	0.000006±0	0.000044±0.000008	2.98	< 0.0001	0.96
Q93099|HGD	0.000047±0.000004	0.00001±0.000002	-1.96	< 0.0001	0.96
Q5JTH9|RRP12	0.000002±0.000001	0.000014±0.000002	6.03	< 0.0001	0.96
Q13228|SBP1	0.00461±0.000275	0.001351±0.000214	-1.81	< 0.0001	0.95
P60903|S10AA	0.000026±0.000004	0.000206±0.00004	3.77	< 0.0001	0.95
Q13636|RAB31	0.00002±0.000001	0.000054±0.000005	1.56	< 0.0001	0.95
P25685|DNJB1	0.000042±0.000002	0.000092±0.00001	1.05	< 0.0001	0.95
Q14764|MVP	0.000152±0.000012	0.000761±0.000127	2.62	< 0.0001	0.95
Q08J23|NSUN2	0.000021±0.000001	0.000048±0.000005	1.15	< 0.0001	0.95
P62847|RS24	0.000026±0.000002	0.0001±0.000011	2.31	< 0.0001	0.95
P43307|SSRA	0.000135±0.000008	0.00037±0.000047	1.48	< 0.0001	0.95
Q9BQG0|MBB1A	0.000016±0.000002	0.00005±0.000005	2.12	< 0.0001	0.95
P05198|IF2A	0.000082±0.000003	0.000143±0.000009	0.83	< 0.0001	0.95

Figures

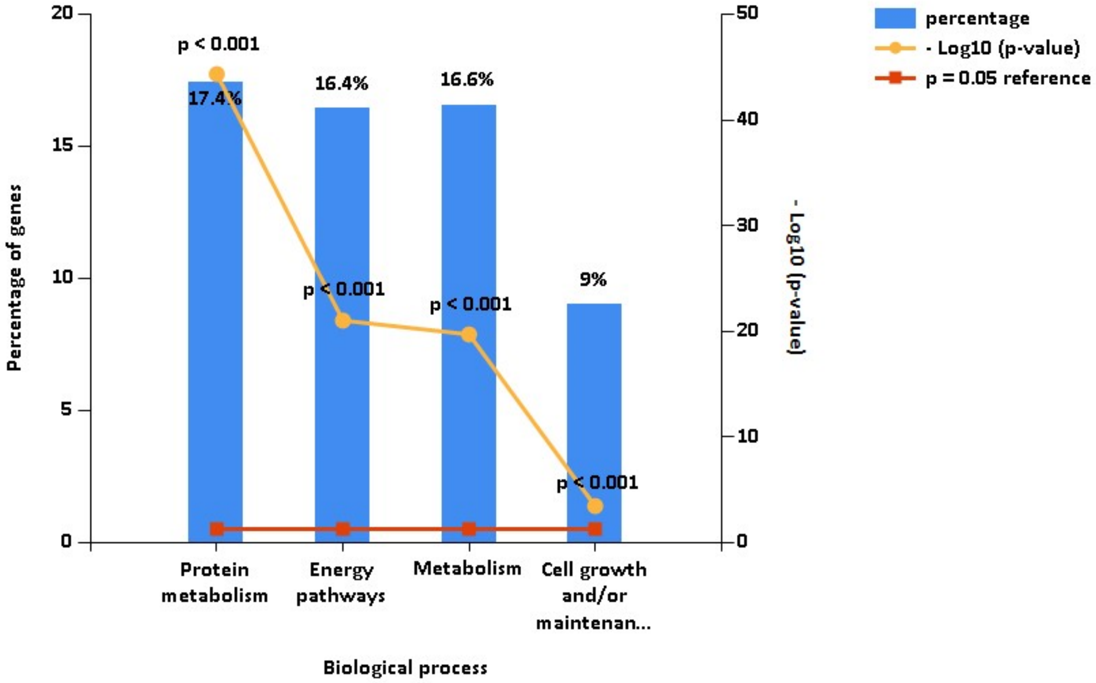

Figure S1. Protein metabolism, energy pathways, intermediary metabolism, and cell growth and/or maintenance are altered in malignant thyroid lesions. Selection of biological processes altered by malignancy with a $p < 0.05$ for the enrichment performed with a hypergeometric test (HG).

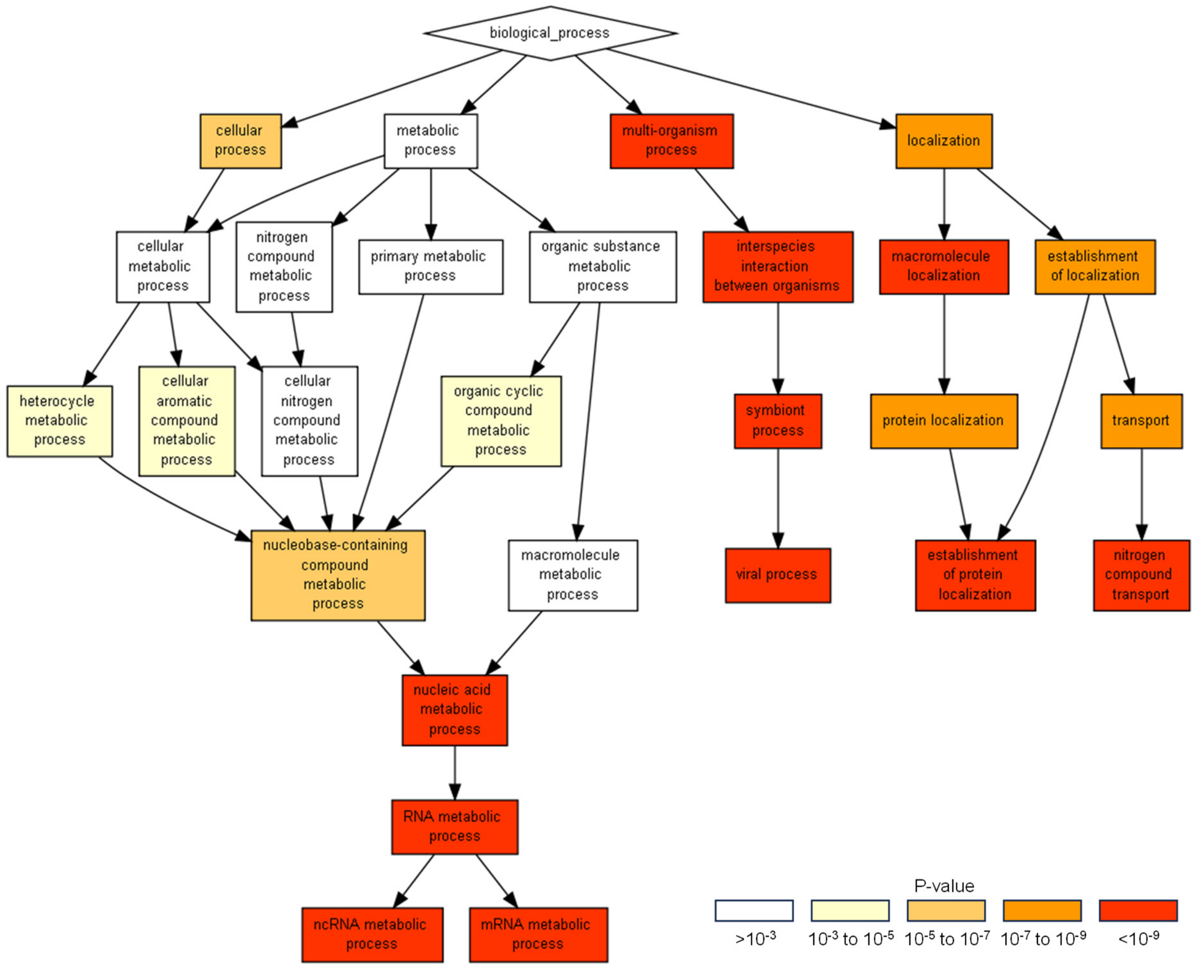

Figure S2. RNA regulation and viral processes are altered in malignant thyroid lesions. Representation of the biological processes altered by malignancy. Only biological processes branches with a $p < 10^{-8}$ are represented. Colour coding reflects the degree of enrichment, with darker red colours representing $p < 10^{-9}$.

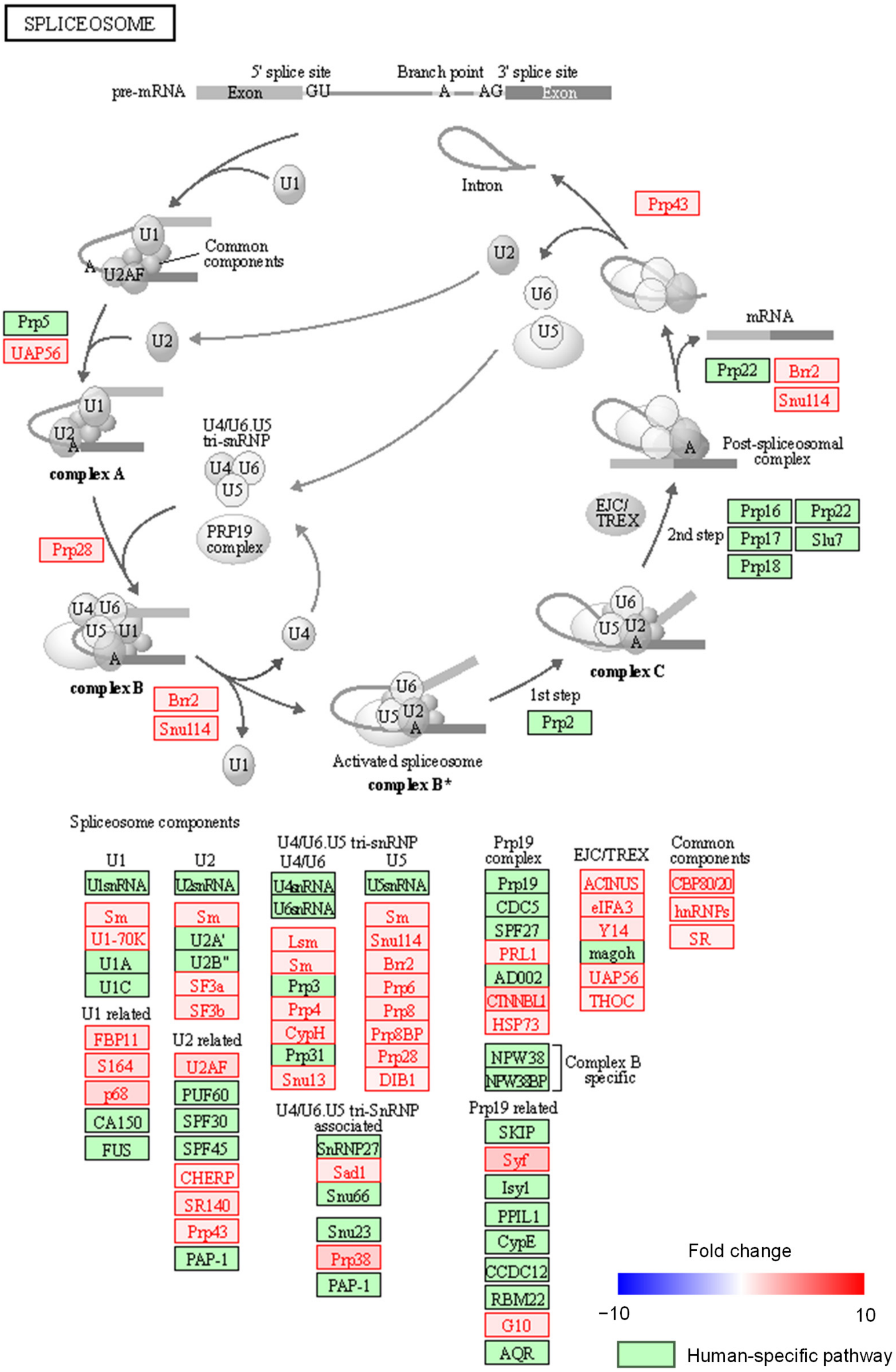

Figure S3. KEGG spliceosome pathway with annotation of statistically altered proteins in malignant thyroid lesions. Upper panel represents main splicing steps and lower panel represents other spliceosome components. Proteins quantified in this study are labelled as red if they are increased in the malignancy group and blue if they are decreased, with this colour code gradient depending on fold change value. Other proteins from human-specific pathways but not quantified in this study are depicted in green.

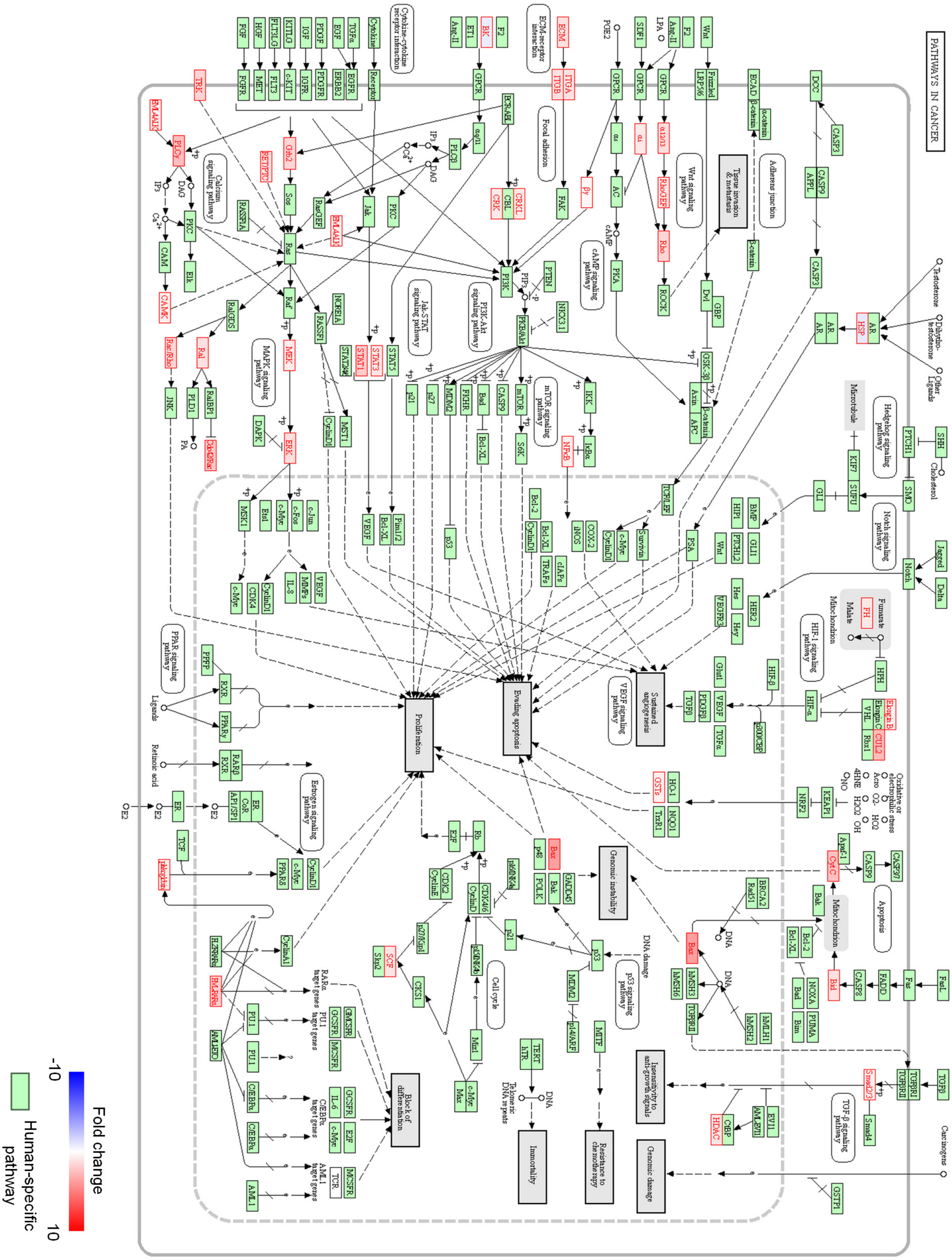

Figure S4. KEGG pathways in cancer with annotation of statistically altered proteins in malignant thyroid lesions. Proteins quantified in this study are labelled as red if they are increased in the malignancy group and blue if they are decreased, with this colour code gradient depending on fold change value. Other proteins from human-specific pathways but not quantified in this study are depicted in green.

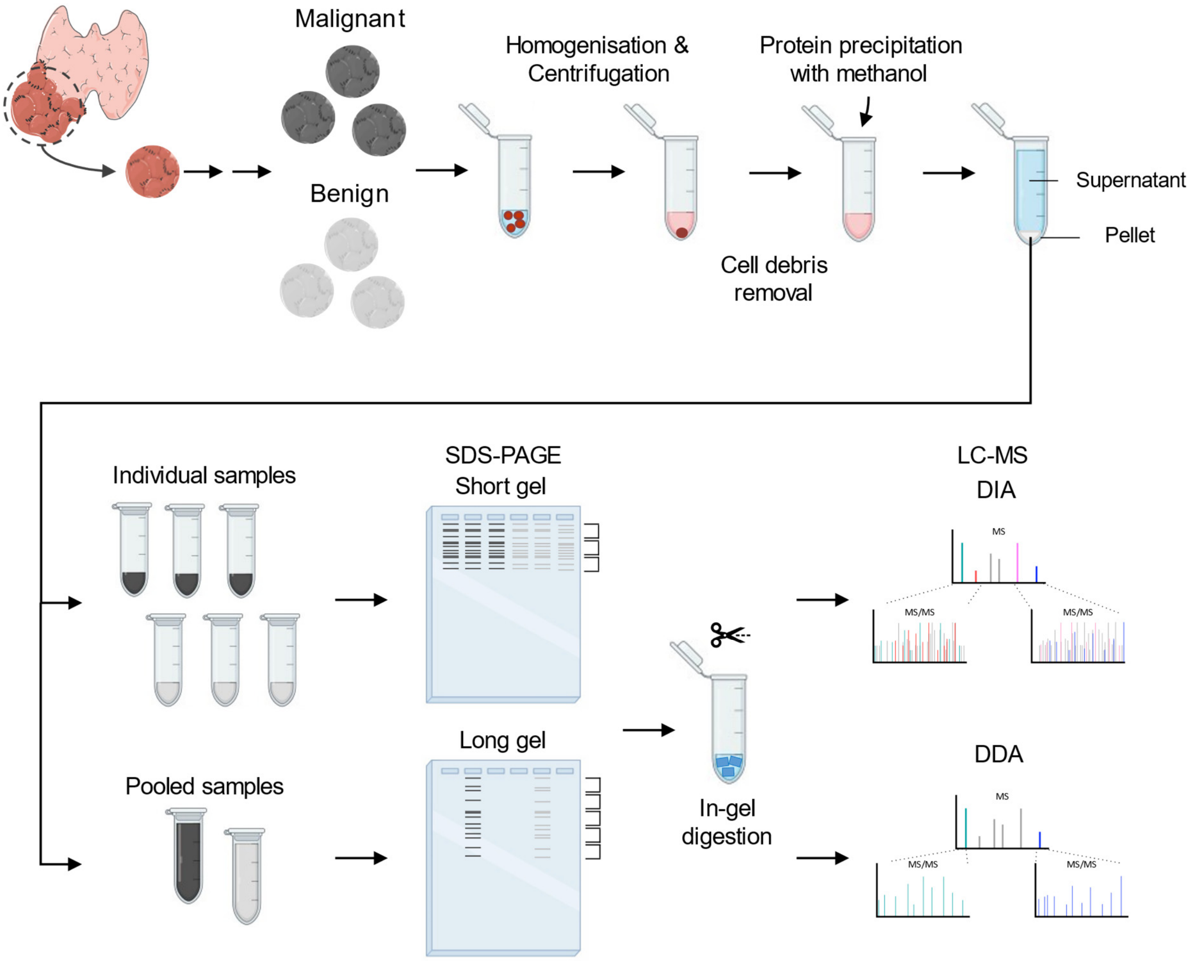

Figure S5. Workflow of thyroid tissues sample preparation for LC-MS proteomics. Malignant samples are represented in black, while benign samples are represented in grey.

References

1. Ali, S.Z.; Baloch, Z.W.; Cochand-Priollet, B.; Schmitt, F.C.; Vielh, P.; VanderLaan, P.A. The 2023 Bethesda System for Reporting Thyroid Cytopathology. *Thyroid* **2023**, *27*, 1341–1346. https://doi.org/10.1089/thy.2023.0141.
2. Lloyd, R.; Osamura, R.; Rosai, J. WHO Classification of Tumours Editorial Board. In *Endocrine and Neuroendocrine Tumours*; IARC: Lyon, France, 2022.
